# Indocyanine green fluorescence imaging as a predictor of long-term testicular atrophy in testicular torsion: a pilot study

**DOI:** 10.1007/s00595-024-02908-9

**Published:** 2024-07-31

**Authors:** Shugo Komatsu, Keita Terui, Ayako Takenouchi, Yunosuke Kawaguchi, Katsuhiro Nishimura, Satoru Oita, Hiroko Yoshizawa, Shota Takiguchi, Tomoro Hishiki

**Affiliations:** Department of Pediatric Surgery, Graduate School of Medicine, Chiba University, 1-8-1 Inohana, Chuo-Ku, Chiba City, Chiba 260-8677 Japan

**Keywords:** Indocyanine green fluorescence, Testicular torsion, Testicular atrophy

## Abstract

**Purpose:**

This pilot study evaluated indocyanine green-guided near-infrared fluorescence (ICG-NIRF) imaging of testicular blood flow to predict long-term testicular atrophy after testicular torsion (TT) surgery.

**Methods:**

The subjects of this retrospective study were patients who underwent surgery for TT at our hospital between December, 2020 and July, 2022. After detorsion, testicular blood flow was evaluated by ICG-NIRF imaging and classified into three categories: fluorescence detected, no fluorescence detected, and fluorescence detected only in the tunica albuginea vessels. Testicular volume was measured by ultrasonography up to 12 months after surgery to evaluate long-term outcomes.

**Results:**

Twelve patients were included in this analysis. We found a 100% correlation between the absence of ICG-NIRF signals and subsequent testicular atrophy. In three patients without an ICG-NIRF signal, the median testis size 12 months postoperatively was significantly smaller (16.5% of the contralateral testis; range 13–20%) than that in six patients with an ICG-NIRF signal (96%; 89–115%) (p = 0.013). Mild atrophy (74.5%; 73–76%) was also observed in the three patients for whom an ICG-NIRF signal was detected only in the tunica albuginea vessels.

**Conclusions:**

Our pilot study highlights the potential of ICG-NIRF imaging as a prognostic tool for guiding surgical decision-making for patients with TT, by predicting postoperative testicular atrophy.

## Introduction

Testicular torsion (TT) is a pediatric surgical emergency that requires prompt diagnosis and intervention to avoid testicular loss. A typical symptom of TT is the sudden onset of intense unilateral testicular pain accompanied by nausea and vomiting [1]. The “golden window of opportunity” to salvage testicular function after onset is within 4–8 h. Failure to intervene within this period results in poor testicular function and a higher rate of orchiectomy [2].

An average of 39% (range, 20–60%) of patients with TT undergo orchiectomies and the rate of testicular loss is reportedly 49–60% [3]. In most cases of TT, intraoperative testicular color observation is used to measure testicular blood flow after detorsion of the testis [1, 4]. Another method to assess testicular blood flow is to look for bleeding following a testicular parenchymal incision [5, 6]. However, there are no established diagnostic standards for testicular preservation during TT surgery. Regardless of which approach is taken, there is no reliable evidence about their efficiency and testicular preservation/removal decisions are strongly influenced by the personal perspectives of the surgeon. Preoperative risk factors such as pain duration > 12 h, heterogeneous testicular parenchyma observed on ultrasonography, and red scrotal changes are related to subsequent testicular atrophy [4]. However, it is important that these characteristics should not be considered as reasons for postponing surgery because some patients do not appear to have complete atrophy.

Indocyanine green (ICG) emits light with a peak at approximately 840 nm when irradiated with near-infrared light (750–810 nm). ICG-guided near-infrared fluorescence (ICG-NIRF) imaging is used widely in various surgical fields such as angiography, sentinel lymph node identification, cholangiography, assessment of vascular supply to organs, and liver cancer surgery [7–12]. ICG-NIRF imaging has also been reported to be useful in the management of TT [13–18]. However, most papers published on this method are case reports, and to our knowledge, there have been no reports of long-term testicular follow-ups.

This pilot study aimed to evaluate the utility of ICG-NIRF imaging for predicting postoperative testicular atrophy after detorsion surgery for TT. By reviewing a cohort of pediatric patients diagnosed with TT who underwent surgical interventions, we aimed to elucidate whether ICG-NIRF imaging findings could serve as a reliable prognostic indicator of testicular viability and subsequent atrophy.

## Methods

### Ethical statement

The study design was approved by the Research Ethics Committee of the Graduate School of Medicine, Chiba University (M10108), and was performed in accordance with the principles of the Declaration of Helsinki and the Ethical Guidelines for Medical and Health Research Involving Human Subjects. The requirement for written informed consent was waived because of the retrospective nature of the study.

### Study design and population

This was a retrospective, single-center cohort study of pediatric patients diagnosed with TT who underwent surgery at our center between December, 2020 and July, 2022. The inclusion criteria were TT confirmed by surgery in patients younger than 15 years old. The exclusion criterion was incomplete hospitalization medical records. Data on background and surgical characteristics were collected from the institutional database. These included age at presentation, affected side, duration of symptoms, rotatory degree of the testis, testicular coloration, ICG-NIRF imaging after detorsion, surgical treatment, and postoperative testicular size.

### Clinical management and assessment of testicular blood flow

**Fig. 1:**
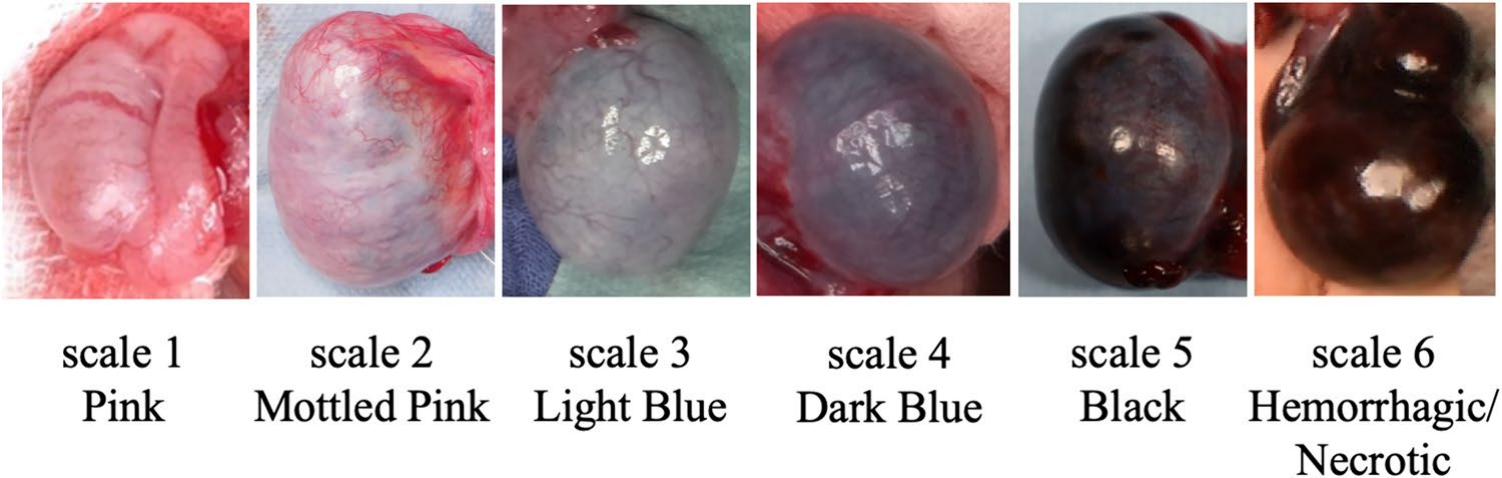
Samples of scale for intraoperative testis color

**Fig. 2:**
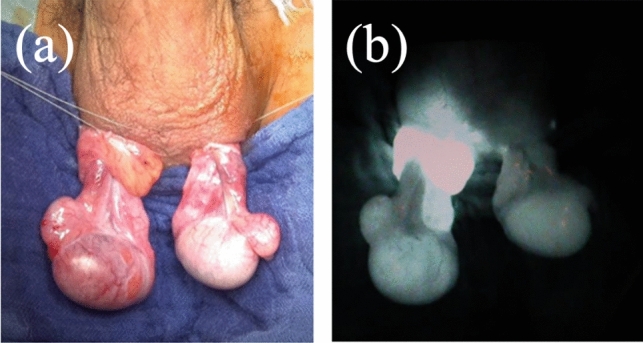
Evaluation of testicular blood flow after the release of right testicular torsion (Patient 2). **a** Macroscopic coloration **b** indocyanine green-guided near-infrared fluorescence imaging

**Fig. 3:**
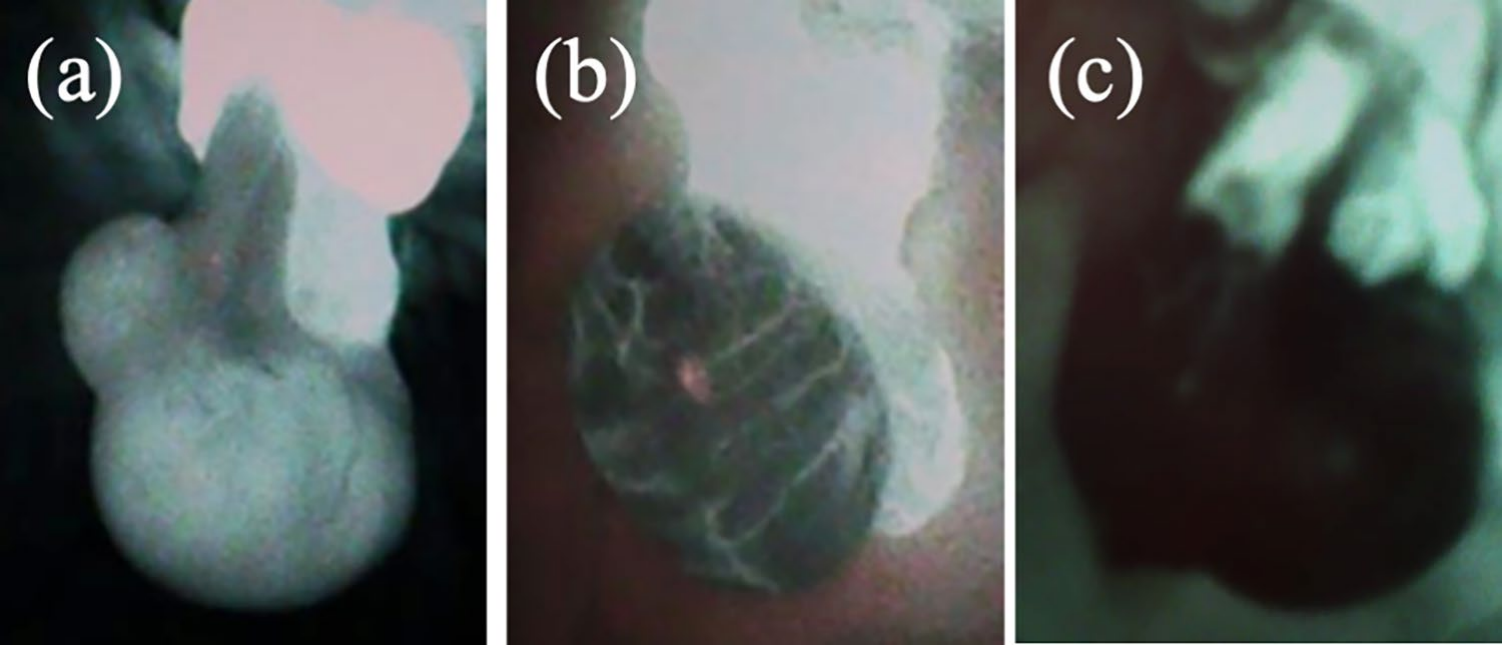
Intraoperative evaluation of testicular blood flow using indocyanine green fluorescence. **a** Pattern A: fluorescence detected in the testicular parenchyma (Patient 2). **b** Pattern B: fluorescence not detected in the testicular parenchyma but only in the tunica albuginea vessels (Patient 8). **c** Pattern C: fluorescence completely negative (Patient 11)

Surgery was performed under general anesthesia. First, the affected testis was explored through a trans-scrotal approach, and the torsion was released. While waiting for blood flow to be restored to the affected testis, we exposed the contralateral testis through a trans-scrotal incision. Once the contralateral testis was exposed, testicular coloration of both testes was reviewed and recorded. Testicular coloration was classified into six categories based on those proposed by Grimsby et al., in which scale 1 represented normal color and scale 6 represented hemorrhage or necrosis (Fig. 1) [4]. Next, we injected 0.1 mg/kg of ICG (Daiichi Sankyo Co., Ltd., Tokyo, Japan) intravenously, and assessed the testicular blood flow using the HyperEye Medical System (Mizuho Medical Co., Ltd.). This system consists of a combination of optical filters and an ultra-high-sensitivity color charge-coupled device image sensor with non-Bayer color filter arrays, which can detect visible light and near-infrared rays from 380 to 1200 nm without color bias at 30 frames per second and can visualize ICG-enhanced structures with vivid color [19]. ICG-NIRF imaging in the affected testis was evaluated simultaneously with the blood flow in the contralateral testis as a control (Fig. 2). We recorded ICG-NIRF imaging in the testes 2 min after administration. Testicular blood flow evaluated using ICG-NIRF imaging was classified into three categories according to the pattern of the fluorescence: pattern A, fluorescence detected in the testicular parenchyma; pattern B, fluorescence not detected in the testicular parenchyma but only in the tunica albuginea vessels; and pattern C, fluorescence completely negative (Fig. 3). The presence or absence of ICG-NIRF imaging was evaluated and recorded. The intensity of ICG-NIRF imaging was not quantified because it could be affected by various inconsistent factors, such as the size and thickness of the testis or the brightness of the room. The decision to preserve or remove the testis was made subjectively by the surgeon based on the color tone after the torsion was released. Since there was no established evidence of the validity of testicular blood flow assessment with ICG-NIRF imaging, the results were not factored into the decision about testis preservation.

Testicular volume was measured using ultrasonography 1, 3, and 12 months postoperatively. Testicular volume was calculated as π/6 × (long diameter) × (short diameter)^2^. Severe and mild testicular atrophies were defined as testicular volume ratios ≤ 50% and 50–80% of the contralateral side, respectively.

### Statistical analysis

Continuous data were compared using the Kruskal–Wallis test with Dunn’s test for multiple comparisons between groups. All statistical analyses were performed using JMP^®^ Pro version 17.0.0 (SAS Institute Inc., Cary, NC, USA). A p-value of < 0.05 was considered significant.

## Results

Twelve patients were included in this analysis. Table 1 summarizes their primary characteristics. The median patient age was 11.5 years (range: 2–14 years). With respect to the laterality of the affected testis, two patients had torsion on the right side and ten had torsion on the left side. The duration of symptoms ranged from 4 to 72 h (median, 7 h), although the time of onset was unclear in one patient. The degree of torsion ranged from 180° to 720° (median, 360°). In all cases, orchidopexy was performed based on the surgeon's subjective judgment of the color tone of the testis after detorsion, and none of the patients underwent orchiectomy. All patients showed favorable tolerance to treatment and were discharged as outpatients. One patient suffered a localized postoperative infection. After determining that testicular sparing was possible, 6 of the 12 patients showed no postoperative testicular atrophy, 3 showed mild atrophy, and 3 showed severe atrophy.

**Table 1:** Patient characteristics

Patient no	Age (years)	Body weight (kg)	Tanner stage	Testicular volume (mL)	Side	Time from onset (hours)
1	11	50	3	9.2	Rt	7
2	12	53	4	15	Rt	4
3	12	45	4	13	Lt	4
4	13	50	4	16	Lt	4
5	14	37	3	9.2	Lt	4
6	14	65	5	19	Lt	5
7	11	37	3	8.1	Lt	9
8	13	35	3	8.3	Lt	8
9	2	14	1	1	Lt	9
10	8	25	1	3.6	Lt	10
11	13	47	4	13	Lt	72
12	5	16	1	1.4	Lt	Unknown

*Lt* left, *Rt* right

Table 2 shows the intraoperative testicular coloration, ICG-NIRF imaging after detorsion, and testicular volume 12 months after surgery. In all patients, ICG-NIRF imaging was observed on the unaffected testis approximately 20 s after ICG administration. ICG-NIRF imaging of the affected testis was detected in 9 of the 12 patients: in the testicular parenchyma (pattern A) in six patients and only in the tunica albuginea vessels (pattern B) in three. ICG-NIRF was negative (pattern C) in the other three patients. No delay in fluorescence detection compared with the unaffected testis was evident in any affected testes showing positive fluorescence. Interoperative testicular color was categorized into Grimsby scale 1 (n = 2), 2 (n = 2), 3 (n = 4), 4 (n = 3), and 5 (n = 1).

**Table 2:** Clinical results

Patient no	Rotation	Testicular coloration scale*	ICG fluorescence pattern**	Surgical treatment	Complications	Testicular volume*** (mL)	Testicular volume ratio**** (%)
1	360°	1	A	Orchiopexy	–	11	115
2	360°	2	A	Orchiopexy	–	16	94
3	360°	2	A	Orchiopexy	–	26	96
4	360°	2	A	Orchiopexy	–	19	89
5	360°	3	A	Orchiopexy	–	14	90
6	540°	3	A	Orchiopexy	–	22	93
7	180°	3	B	Orchiopexy	–	7.1	76
8	360°	3	B	Orchiopexy	–	5	73
9	360°	4	B	Orchiopexy	–	0.5	69
10	360°	4	C	Orchiopexy	–	0.2	20
11	360°	4	C	Orchiopexy	–	0.4	13
12	720°	5	C	Orchiopexy	Wound infection	0.3	6

*Scale 1, pink; scale 2, mottled pink; scale 3, light blue; scale 4, dark blue; scale 5, black

**Pattern A: fluorescence detected in the testicular parenchyma. Pattern B: fluorescence not detected in the testicular parenchyma but only in the tunica albuginea vessels. Pattern C: fluorescence completely negative

***Affected testicular volume at 12 months

****Relative size (%) of the affected testis compared with the contralateral testis at 12 months

**Fig. 4:**
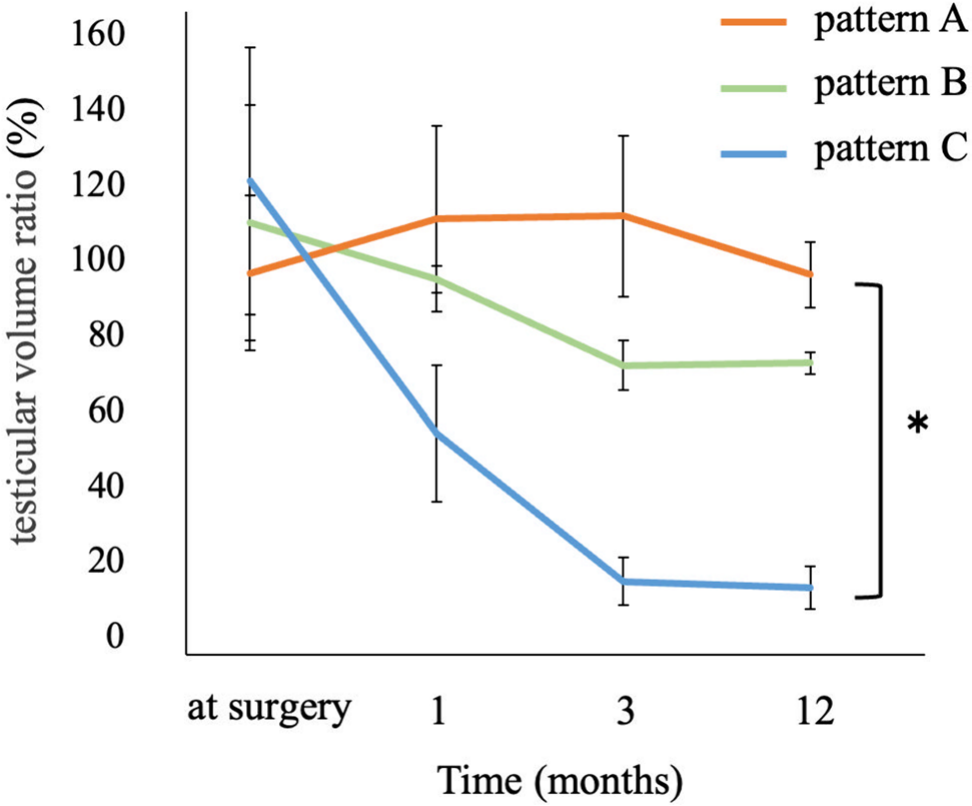
Testicular coloration and indocyanine green fluorescence after detorsion and testicular volume at 12 months of follow-up. Scale 1, pink; scale 2, mottled pink; scale 3, light blue; scale 4, dark blue; scale 5, black. Pattern A, fluorescence detected in the testicular parenchyma; B fluorescence not detected in the testicular parenchyma but only in the tunica albuginea vessels; C fluorescence completely negative. The testicular volume ratio indicates the relative size (%) of the affected testis compared with the contralateral testis. *The testis size in pattern **C** was significantly smaller than that in pattern **A** (*p* = 0.013)

Postoperative evaluations 12 months after surgery revealed that the testicular volume ratio was > 80% in patients 1–6, with no atrophy observed. In contrast, it was < 20% in patients 10–12, with severe atrophy observed, and 60–80% in patients 7–9, with mild atrophy observed. Postoperative testicular atrophy did not develop in the four patients whose affected testes were categorized as color scale 1 or 2; however, the patient whose testis color was categorized into scale 5 showed strong postoperative testicular atrophy. The four patients with scale 3 testes had no (n = 2) or mild (n = 2) atrophy, whereas the three patients in the scale 4 group had mild (n = 1) or severe (n = 2) testicular atrophy (Fig. 4).

**Fig. 5:**
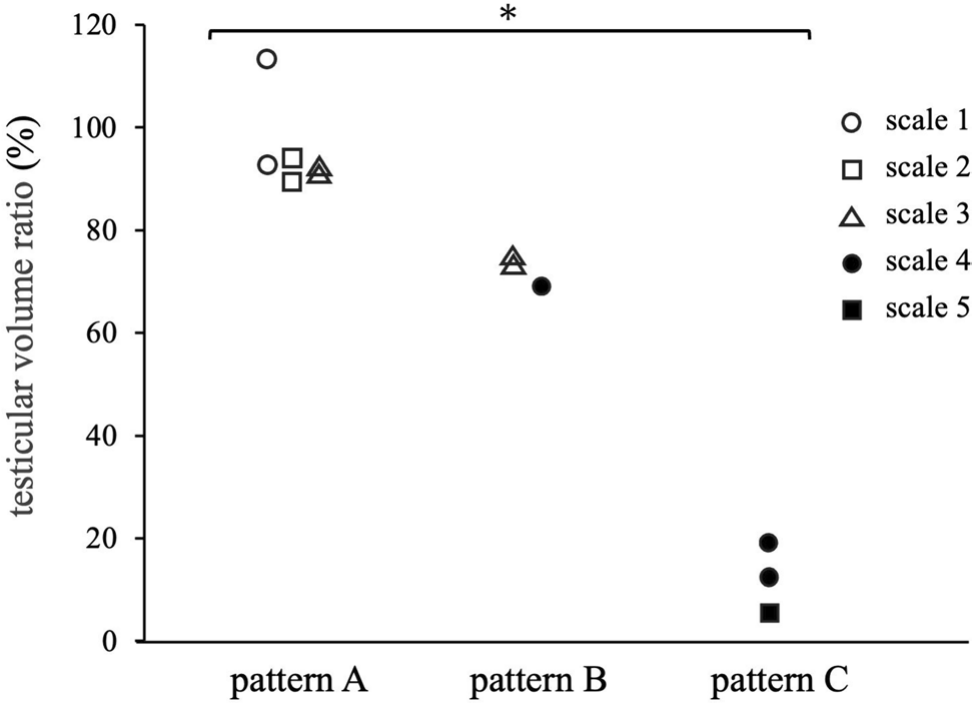
Intraoperative indocyanine green fluorescence in the testis and postoperative change in testicular volume. The testicular volume ratio indicates the relative size (%) of the affected testis compared with the contralateral testis. *The testis size in pattern C was significantly smaller than that in pattern A (p = 0.013)

The postoperative testicular volume correlated with the findings from the ICG-NIRF imaging assessment. No atrophy was observed in patients with pattern A, mild atrophy was observed in those with pattern B, and severe atrophy was observed in those with pattern C. Figure 5 shows the changes in the testicular volume ratio over time after surgery for each ICG-NIRF imaging pattern. Testes with pattern C showed severe atrophy as early as 1 month postoperatively. In pattern B, mild atrophy became evident 3 months postoperatively, but the testicular volume ratio remained unchanged thereafter for up to 12 months. The median testicular volume ratio 12 months postoperatively was 16.5% (range; 13–20%) in pattern C, 74.5% (73–76%) in pattern B, and 96% (89–115%) in pattern A. The testicular volume ratio in pattern C was significantly smaller than that of pattern A, 12 months postoperatively (p = 0.013).

## Discussion

This pilot study underscores the potential of ICG-NIRF imaging as a reliable prognostic tool for assessing testicular viability and predicting subsequent atrophy following detorsion surgery for TT. The integration of ICG-NIRF imaging into routine practice has the potential to improve the surgical decision-making about whether the affected testis should be removed. The current standard is to attempt to preserve as much of the testis as possible; however, it may be equally important to properly resect testes that are already necrotic. Preserving a potentially non-viable testicle can cause issues such as pain and postoperative infection [4, 20]. By providing real-time visualization of the testicular blood flow, surgeons can assess the viability of the affected testis more confidently and individualize intraoperative management accordingly. Traditional methods for assessing testicular viability during surgery, such as intraoperative coloration and ultrasound findings, lack standardization and may be subject to interpretation bias [21, 22]. Thus, exploring novel techniques, such as ICG-NIRF imaging, could improve the accuracy of intraoperative assessments and predict long-term postoperative outcomes.

Although none of the patients in this study underwent a testicular fasciotomy at the discretion of the surgeon, testicular fasciotomy may be appropriate for the treatment of vascularly impaired testes [23, 24]. Evaluation by ICG-NIRF imaging could help determine the indication for testicular fasciotomy. Even if the testis appears well colored, if there is no ICG fluorescence of the testis, the testis is likely to atrophy postoperatively even if it is fixed as is. Therefore, in the absence of ICG fluorescence of the testis, a testicular fasciotomy should be seriously considered. Moreover, it would be important to follow up the testicular prognosis of cases in which blood flow by ICG-NIRF imaging was restored by testicular fasciotomy.

The strength of this study was that none of the patients underwent orchiectomy and all patients were followed up for testicular preservation. The current findings demonstrated a close correlation between ICG-NIRF imaging findings and postoperative testicular outcomes (Fig. 5). ICG-NIRF imaging was able to predict testicular atrophies that were unpredictable based on testicular coloration (Fig. 4). For testes with color scale 3 or 4, it was difficult to predict testicular atrophy based on coloration alone because testicular atrophy developed after surgery in some patients but not in others. All patients whose parenchyma of the affected testis were negative for ICG-NIRF imaging (patterns B and C) suffered mild or severe postoperative testicular atrophy, whereas testicular atrophy was not observed in any patient in whom ICG-NIRF imaging was detected in the parenchyma of the affected testis (pattern A). Notably, patients with negative ICG-NIRF imaging in the parenchyma but detectable signs in the tunica albuginea vessels (pattern B) suffered mild but not severe atrophy of the affected testis. The median period from surgery to testicular atrophy is reportedly 12.5 months [25]; therefore, it is necessary to continue monitoring for testicular atrophy and consider the advantages and disadvantages of testicular preservation in these patients.

There are two types of commercial detectors for near-infrared fluorescence devices: handles and endoscopes. The handle type excites fluorescence more intensely than the endoscopic type, even in the same lesion. The choice of the device is essential: the handle type is generally used in open abdominal and thoracic surgeries, whereas the endoscopic type is more often used in endoscopic surgery [26]. Although endoscopic camera systems using 4 K full-color plus overlay and near-infrared optical imaging technology evaluate testicular blood flow [15–18], we used a monochrome type intentionally because the fluorescence intensity in the contralateral testis would be easier to compare.

In our pediatric patients, ICG signals became visible approximately 20 s after intravenous ICG injection. We recorded the strongest luminescence during the first 3 min after injection; however, there were no criteria for the ICG appearance time to evaluate blood flow in the TT. Shirasaki et al. [15] reported that the testis could be preserved if ICG signals appeared within 90 s. The ICG signal was confirmed within 90 s in all our patients with testicular blood flow confirmed, and there were no cases of atrophy. The optimal dosage of ICG for pediatric patients remains undetermined, although while previous studies have reported administering intravenous ICG 0.2–0.3 mg/kg [15, 17], we found that an intravenous dose of 0.1 mg/kg was adequate for assessing testicular blood flow. Given the limited number of studies on the use of ICG to evaluate blood flow in children, further studies are needed to develop standardized and validated protocols specific to TT imaging. The potential adverse effects of ICG include anaphylactic shock, hypotension, tachycardia, dyspnea, and urticaria [27]. However, these side effects are uncommon, and the incidence of adverse reactions is less than 0.01% [26].

Some studies have reported that ultrasonographic findings indicating decreased testicular blood flow were not predictive of testicular atrophy [28, 29]. The present study did not directly compare ICG-NIRF imaging findings with Doppler ultrasonography. While the current study highlights the potential of ICG-NIRF imaging, further research is needed to confirm its superiority over ultrasonographic assessment.

Although this study provides valuable insights into the potential utility of ICG-NIRF imaging in TT surgery, it has some limitations. First, it was a preliminary/pilot study with a small sample size, limiting the generalizability and statistical power of the findings. There is also potential bias due to the retrospective nature and single-center design. Prospective studies using a larger cohort are warranted to validate our results and to further elucidate the role of ICG-NIRF imaging in TT management. Additionally, the observation period was relatively short because of follow-up constraints. Evaluation of functional outcomes such as fertility and hormone levels based on long-term follow-up may yield further valuable findings.

In conclusion, this pilot study highlights the promising role of ICG-NIRF imaging as a predictive tool for TT by providing an objective assessment of testicular perfusion and guiding surgical decision-making. ICG-NIRF imaging may help prevent the removal of functional testes and preserve necrotic testes during TT surgery. Further research is warranted to validate these findings and optimize the integration of ICG-NIRF imaging in clinical practice, ultimately improving the care and outcomes of patients with TT.

## References

[1] Sharp VJ, Kieran K, Arlen AM. Testicular torsion: diagnosis, evaluation, and management. Am Fam Physician. 2013;88:835–40. 24364548

[2] Bowlin PR, Gatti JM, Murphy JP. Pediatric testicular torsion. Surg Clin North Am. 2017;97:161–72. 27894425 10.1016/j.suc.2016.08.012

[3] MacDonald C, Kronfli R, Carachi R, O’Toole S. A systematic review and meta-analysis revealing realistic outcomes following paediatric torsion of testes. J Pediatr Urol. 2018;14:503–9. 30404723 10.1016/j.jpurol.2018.09.017

[4] Grimsby GM, Schlomer BJ, Menon VS, Ostrov L, Keays M, Sheth KR, et al. Prospective evaluation of predictors of testis atrophy after surgery for testis torsion in children. Urology. 2018;116:150–5. 29572055 10.1016/j.urology.2018.03.009 PMC6291205

[5] Cimador M, DiPace MR, Castagnetti M, DeGrazia E. Predictors of testicular viability in testicular torsion. J Pediatr Urol. 2007;3:387–90. 18947779 10.1016/j.jpurol.2007.01.194

[6] Arda IS, Ozyaylali I. Testicular tissue bleeding as an indicator of gonadal salvageability in testicular torsion surgery. BJU Int. 2001;87:89–92. 11121999 10.1046/j.1464-410x.2001.00021.x

[7] Aoun F, Albisinni S, Zanaty M, Hassan T, Janetschek G, van Velthoven R. Indocyanine green fluorescence-guided sentinel lymph node identification in urologic cancers: a systematic review and meta-analysis. Minerva Urol Nefrol. 2018;70:361–9. 29241310 10.23736/S0393-2249.17.02932-0

[8] Oldhafer KJ, Reese T, Fard-Aghaie M, Strohmaier A, Makridis G, Kantas A, et al. Intraoperative fluorescence angiography and cholangiography with indocyanine green in hepatobiliary surgery. Chirurg. 2019;90:880–6. 31559461 10.1007/s00104-019-01035-3

[9] Esposito C, Settimi A, Del Conte F, Cerulo M, Coppola V, Farina A, et al. Image-guided pediatric surgery using indocyanine green (ICG) fluorescence in laparoscopic and robotic surgery. Front Pediatr. 2020;8:314. 32626676 10.3389/fped.2020.00314 PMC7311575

[10] Namikawa T, Sato T, Hanazaki K. Recent advances in near-infrared fluorescence-guided imaging surgery using indocyanine green. Surg Today. 2015;45:1467–74. 25820596 10.1007/s00595-015-1158-7

[11] Yamada Y, Ohno M, Fujino A, Kanamori Y, Irie R, Yoshioka T, et al. Fluorescence-guided surgery for hepatoblastoma with indocyanine green. Cancers (Basel). 2019;11:1215. 31434361 10.3390/cancers11081215 PMC6721588

[12] Komatsu S, Terui K, Nakata M, Shibata R, Oita S, Kawaguchi Y, et al. Combined use of three-dimensional construction and indocyanine green-fluorescent imaging for resection of multiple lung metastases in hepatoblastoma. Children (Basel). 2022;9:376. 35327748 10.3390/children9030376 PMC8947451

[13] Kohler R, Hamdani A, Grämiger M. Use of intraoperative Indocyanine green fluorescence to assess testicular perfusion and viability when managing testicular torsion in a 26-year old man. Urol Case Rep. 2020;28: 101063. 31754602 10.1016/j.eucr.2019.101063 PMC6854066

[14] Şencan A, Tanriverdi HI, Şimşek FB, Usta İB, Üçöz M, Özbilgin K. Intraoperative evaluation of testicular vascularization and perfusion in rat testicles with indocyanine green (ICG)/near-infrared (NIR) fluorescent imaging after torsion-detorsion and reperfusion. Pediatr Surg Int. 2022;38:1625–33. 36087144 10.1007/s00383-022-05211-1

[15] Shirasaki Y, Kawashima M, Kimura T, Yamanaka H, Hatta K, Branch J, et al. Successful salvage of torsion testis by means of intraoperative indocyanine green fluorescence imaging. Surg Case Rep. 2022;8:152. 35951275 10.1186/s40792-022-01476-9 PMC9372249

[16] Liu X, Xu Y, Li L, Bai D. Evaluation of testicular blood flow during testicular torsion surgery in children using the indocyanine green-guided near-infrared fluorescence imaging technique. Front Pediatr. 2023;11:1272659. 37964816 10.3389/fped.2023.1272659 PMC10642505

[17] Kameoka Y, Yoshimura S, Matsufuji H, Umeyama T, Machigashira S, Yada K. Criteria for preserving grossly ischemic torsed testicles using indocyanine green fluorescence imaging: a single-center case series. Urology. 2023;178:133–7. 37030579 10.1016/j.urology.2022.11.061

[18] Savoie-White FH, Mailloux O. Use of intraoperative indocyanine green fluorescence in determining testicular viability in testicular torsion patients in rural settings: a case report. Int J Surg Case Rep. 2023;106: 108247. 37087930 10.1016/j.ijscr.2023.108247 PMC10149194

[19] Handa T, Katare RG, Sasaguri S, Sato T. Preliminary experience for the evaluation of the intraoperative graft patency with real color charge-coupled device camera system: an advanced device for simultaneous capturing of color and near-infrared images during coronary artery bypass graft. Interact Cardiovasc Thorac Surg. 2009;9:150–4. 19423513 10.1510/icvts.2008.201418

[20] Jacobsen FM, Rudlang TM, Fode M, Østergren PB, Sønksen J, Ohl DA, et al. The impact of testicular torsion on testicular function. World J Mens Health. 2020;38:298–307. 31081295 10.5534/wjmh.190037 PMC7308234

[21] Hyun GS. Testicular torsion. Rev Urol. 2018;20:104–6. 30288149 10.3909/riu0800 PMC6168322

[22] Wang JH. Testicular torsion. Urol Sci. 2012;23:85–6.

[23] Kutikov A, Casale P, White MA, Meyer WA, Chang A, Gosalbez R, et al. Testicular compartment syndrome: a new approach to conceptualizing and managing testicular torsion. Urology. 2008;72:786–9. 18561988 10.1016/j.urology.2008.03.031

[24] Figueroa V, Pippi Salle JL, Braga LH, Romao R, Koyle MA, Bägli DJ, et al. Comparative analysis of detorsion alone versus detorsion and tunica albuginea decompression (fasciotomy) with tunica vaginalis flap coverage in the surgical management of prolonged testicular ischemia. J Urol. 2012;188:1417–22. 22906680 10.1016/j.juro.2012.02.017

[25] Lian BS, Ong CC, Chiang LW, Rai R, Nah SA. Factors predicting testicular atrophy after testicular salvage following torsion. Eur J Pediatr Surg. 2016;26:17–21. 26509312 10.1055/s-0035-1566096

[26] Chu DI, Gupta K, Kawal T, Van Batavia JP, Bowen DK, Zaontz MR, et al. Tunica vaginalis flap for salvaging testicular torsion: a matched cohort analysis. J Pediatr Urol. 2018;14:329.e1-329.e7. 29454628 10.1016/j.jpurol.2018.01.010 PMC6078825

[27] Bašković M. Letter to the Editor in reference to the article entitled ‘Intraoperative evaluation of testicular vascularization and perfusion in rat testicles with indocyanine green (ICG)/near-infrared (NIR) fluorescent imaging after torsion-detorsion and reperfusion.’ Pediatr Surg Int. 2022;39:1. 36434473 10.1007/s00383-022-05279-9

[28] Hiyama E. Fluorescence image-guided navigation surgery using indocyanine green for hepatoblastoma. Children (Basel). 2021;8:1015. 34828728 10.3390/children8111015 PMC8617810

[29] Tian XM, Tan XH, Shi QL, Wen S, Lu P, Liu X, et al. Risk factors for testicular atrophy in children with testicular torsion following emergent orchiopexy. Front Pediatr. 2020;8: 584796. 33262963 10.3389/fped.2020.584796 PMC7686235

